# The Genomic Architecture of Competitive Response of *Arabidopsis thaliana* Is Highly Flexible Among Plurispecific Neighborhoods

**DOI:** 10.3389/fpls.2021.741122

**Published:** 2021-11-25

**Authors:** Cyril Libourel, Etienne Baron, Juliana Lenglet, Laurent Amsellem, Dominique Roby, Fabrice Roux

**Affiliations:** ^1^Laboratoire des Interactions Plantes-Microbes-Environnement, Institut National de Recherche pour l’Agriculture, l’Alimentation et l’Environnement, CNRS, Université de Toulouse, Castanet-Tolosan, France; ^2^Laboratoire Evolution, Ecologie et Paléontologie, UMR CNRS 8198, Université de Lille, Villeneuve d’Ascq Cedex, France

**Keywords:** plant-plant interactions, *Arabidopsis thaliana*, genetic variation, GWAS, local population, plurispecific interactions

## Abstract

Plants are daily challenged by multiple abiotic and biotic stresses. A major biotic constraint corresponds to competition with other plant species. Although plants simultaneously interact with multiple neighboring species throughout their life cycle, there is still very limited information about the genetics of the competitive response in the context of plurispecific interactions. Using a local mapping population of *Arabidopsis thaliana*, we set up a genome wide association study (GWAS) to estimate the extent of genetic variation of competitive response in 12 plant species assemblages, based on three competitor species (*Poa annua*, *Stellaria media*, and *Veronica arvensis*). Based on five phenotypic traits, we detected strong crossing reaction norms not only between the three bispecific neighborhoods but also among the plurispecific neighborhoods. The genetic architecture of competitive response was highly dependent on the identity and the relative abundance of the neighboring species. In addition, most of the enriched biological processes underlying competitive responses largely differ among neighborhoods. While the RNA related processes might confer a broad range response toolkit for multiple traits in diverse neighborhoods, some processes, such as signaling and transport, might play a specific role in particular assemblages. Altogether, our results suggest that plants can integrate and respond to different species assemblages depending on the identity and number of each neighboring species, through a large range of candidate genes associated with diverse and unexpected processes leading to developmental and stress responses.

## Introduction

Plant-plant interactions are recognized as a major factor mediating the plant community structure, diversity, and dynamics (Tilman, 1985; Goldberg and Barton, 1992; Chesson, 2000; Martorell and Freckleton, 2014). Thus, deciphering the genetic and molecular bases of plant-plant interactions appears fundamental to predicting the evolutionary dynamics of plant communities in ecological time (Pierik et al., 2013; Frachon et al., 2017). This is especially relevant in the context of current anthropogenic modifications of plant assemblages, which may in part result from the intertwined effect of increased plant biomass and reduced plant diversity under climate warming (Baldwin et al., 2014) or from the native species having different geographical range shifts under climate change (Bachelet et al., 2001; Gilman et al., 2010; Singer et al., 2013). Furthermore, in the absence of pesticides, the reduction in crop yield by weeds is significantly higher than by any other crop pests (Oerke, 2006; Neve et al., 2009). Identifying and characterizing the function of genes underlying crop-weed interactions appears therefore fundamental to accelerate the breeding programs aimed at regulating the weeds (Worthington and Reberg-Horton, 2013; Onishi et al., 2018). In addition, in the context of complementarity in using resources, optimizing species assemblages in the crops may be facilitated by the understanding of the genetics underlying overyielding (Litrico and Violle, 2015; Pakeman et al., 2015; Weiner et al., 2017).

In comparison with other types of biotic interactions, such as plant response to virus, bacteria, fungi, oomycetes, and to a lesser extent, herbivores (Roux and Bergelson, 2016; Bartoli and Roux, 2017), there is still very limited information about the genetics associated with natural variation of plant-plant interactions, i.e., when plants are directly challenged by other plants. For example, a recent review listed only 47 quantitative trait loci (QTL) mapping studies (including, three genome wide association studies, GWAS) that have been designed to study the genetic architecture underlying natural variation of plant-plant interactions (Subrahmaniam et al., 2018). Natural genetic variation in plant-plant interactions is mainly driven by a complex genetic architecture, ranging from the identification of few medium-effect QTLs to the identification of up to tens of small-effect QTLs (Subrahmaniam et al., 2018). In a heterospecific context, more than 80% of the QTL mapping studies focused on asymmetric interactions (i.e., when one of the interacting partners benefits at the expense of the other), including response to the parasitic plants and weed suppressive ability mediated by allelopathy (Subrahmaniam et al., 2018). Surprisingly, despite the importance of competition in driving plant community assemblages, only six QTL mapping studies (including two GWAS) focused on the competitive interactions in a heterospecific context, i.e., when both the interacting species suffer significant cost by investing in competing and therefore compromising on the benefit (Dudley, 2015). Recently, we reported the results of a GWAS focusing on bispecific heterospecific interactions (i.e., single pair of interacting species; Baron et al., 2015). In this GWAS, we used 48 natural accessions of *Arabidopsis thaliana* from the highly genetically polymorphic French TOU-A local population located in a highly competitive habitat (Frachon et al., 2017) and genotyped with a single nucleotide polymorphism (SNP)-array of 214,051 SNPs (Hancock et al., 2011; Horton et al., 2012). These accessions were grown in field conditions with four competitor species frequently associated with *A. thaliana* in natural plant communities (i.e., *Poa annua*, *Stellaria media*, *Trifolium arvense*, and *Veronica arvensis*). Interestingly, the genetic architecture was found to be highly flexible between the four competitor species for each of the nine phenotypic traits measured on the *A. thaliana* plants, including a proxy of total seed production (Baron et al., 2015).

Most of the QTL mapping studies conducted in a heterospecific context were based on bispecific interactions (Subrahmaniam et al., 2018). However, throughout their life cycle, the focal plants often interact simultaneously with several neighboring species, either in crop fields or in natural communities (Wilson et al., 2012). This highlights the need to study the genetic architecture underlying plant-plant interactions by considering the response of a focal species to the plurispecific interactions. In particular, whether the genetic architecture underlying the response of a focal species in a plurispecific neighborhood corresponds to the sum of QTLs that are specific to the presence of a neighboring species and/or to the emergence of new QTLs, remains on an open question (Subrahmaniam et al., 2018). In a first attempt to address this question, a Genome-Environment Association (GEA) analysis was recently set up to finely map the genomic regions of *A. thaliana* associated with various plant community descriptors in 145 natural populations of *A. thaliana* located in the south-west of France (alpha-diversity, community composition, and the presence/absence of the 44 most prevalent plant species; Frachon et al., 2019). The genetic architecture was highly dependent not only on the identity of a specific neighboring species but also on the diversity and composition of the plant communities inhabited by *A. thaliana* (Frachon et al., 2019). In agreement, the candidate genes underlying the QTLs were highly dependent on the assemblage of plant species co-occurring with *A. thaliana* (Methods in Supplementary Information, Supplementary Figure 1), suggesting a flexible genetic architecture between bispecific and plurispecific plant-plant interactions. The main drawback of GEA studies performed on biotic descriptors is, however, related to the confounding effects of abiotic factors, such as climate variables and soil physicochemical properties, thereby advocating the need for a controlled common garden approach to confirm or not the main conclusions obtained from a GEA approach (Frachon et al., 2019).

We extended the study from Baron et al. (2015) by setting up a GWAS to compare the genetic architecture of competitive response of *A. thaliana* between bispecific and plurispecific neighborhoods. Based on a common garden experiment performed in greenhouse conditions, we first estimated the extent of genetic variation of competitive response in a set of 91 whole-genome sequenced *A. thaliana* accessions from the same French TOU-A local population previously used in Baron et al. (2015). These accessions were submitted to bispecific and plurispecific competition treatments based on all one-way, two-way, and three-way combinations of *P. annua*, *S. media*, and *V. arvensis*. Based on the whole-genome sequences of the 91 accessions, we then, run genome-wide association (GWA) mapping combined with a local score approach to compare the genetic architecture of competitive response of *A. thaliana* between the bispecific and plurispecific neighborhoods. Finally, we compared the biological processes over-represented among the candidate genes identified in competitive response among the bispecific and plurispecific neighborhoods and discussed the function of some candidate genes.

## Materials and Methods

### Plant Material

A set of 96 whole-genome sequenced accessions of *A. thaliana* collected in the TOU-A local population (Toulon-sur-Arroux, Burgundy, France, 46°38′53.80″N–4° 7′22.65″E) was used for the purpose of this study. As previously described in Frachon et al. (2017), the TOU-A population is highly polymorphic at both the phenotypic and genomic levels. Importantly, the very short linkage disequilibrium (LD) observed in this population ($r_{0.5}^{2}$∼18bp) allows the fine mapping of genomic regions associated with natural variation of phenotypic traits down to the gene level (Brachi et al., 2013; Huard-Chauveau et al., 2013; Frachon et al., 2017; Aoun et al., 2020; Lonjon et al., 2020). These 96 accessions were chosen among a set of 195 accessions to be representative of the genetic diversity observed in the TOU-A population (Platt et al., 2010).

The maternal effects of the 96 accessions were reduced by growing one plant of each family for one generation under controlled greenhouse conditions (16-h photoperiod and 20°C) in early 2011 at the University of Lille, Lille, France. Given an estimated selfing rate of ∼94% in this population (Platt et al., 2010), the 96 accessions were considered as mostly homozygous along the genome.

In this study, we used three neighboring species commonly associated with *A. thaliana* in natural plant communities in France and detected in the TOU-A plant community (F. Roux, personal observation). These species are the meadow grass *P. annua* (Poaceae) with a low spreading growth form, the chickweed *S. media* (Caryophyllaceae), and the speedwell *V. arvensis* (Scrophulariaceae) both with a crawling growth form. The seeds for these three species were obtained from the Herbiseeds company.^1^

### Phenotypic Characterization

#### Experimental Design

**FIGURE 1:**
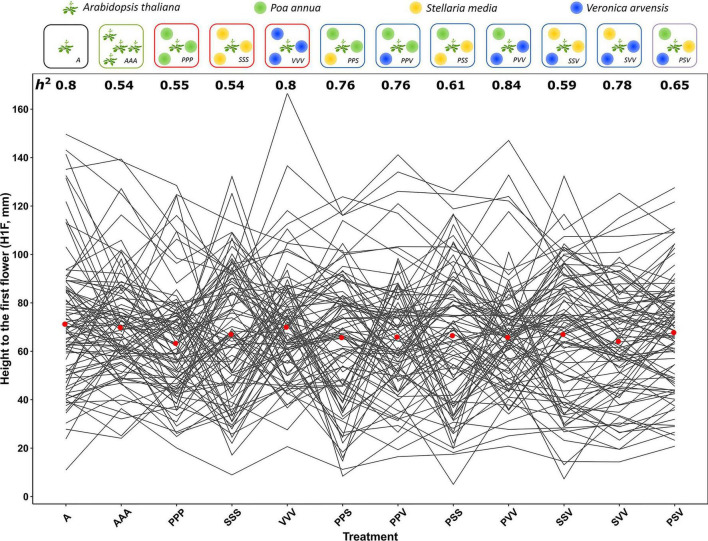
Natural genetic variation of reactions norms of 89 TOU-A accessions across 12 plant–plant interaction treatments. **(Top panel)** Diagram illustrating the 12 treatments. **(Bottom panel)** Height from the soil to the first flower on *Arabidopsis thaliana* (H1F). Each line links the genotypic values of one of 89 TOU-A accessions. The two remaining accessions A1-69 and A1-117 are not represented due to the missing genotypic values in the PPV and SSS treatments, respectively. For a given treatment, the mean H1F genotypic value among the accessions is represented by a red dot. *h*^2^: narrow-sense heritability estimates.

An experiment of 4,608 focal plants of *A. thaliana* and 12,672 neighboring plants was set up at the University of Lille (France), using a split-plot design arranged as a randomized complete block design (RCBD) with 12 treatments nested within four blocks. These 12 competition treatments correspond to (Figure 1):

– One control treatment where *A. thaliana* was grown alone (i.e., absence of interaction; hereafter named treatment A).
– The 10 interspecific interaction treatments corresponding to the full combination of the three neighboring species *P. annua* (P), *S. media* (S), and *V. arvensis* (V): PPP, SSS, VVV, PPS, PPV, PSS, PVV, SSV, SVV, and PSV.
– One intraspecific interaction treatment (hereafter named treatment AAA). This treatment was included in the experiment to test whether the differences observed between the treatment where *A. thaliana* was grown alone (i.e., treatment A) and the treatments of interspecific interactions were not only due to the presence of a neighboring plant but were rather dependent on either the identity of the neighboring species or the combination of neighboring species. With respect to the barochorous mode of seed dispersal in *A. thaliana* (Wender et al., 2005; Weinig et al., 2006), the intraspecific interaction treatment corresponds to an intra-genotypic interaction.

Each “block × competition treatment” combination was represented by 96 pots (7 cm × 7 cm × 7 cm, vol. ∼ 250 cm^3^; TEKU MQC) filled with damp standard culture soil (Huminsubstrat N3, Neuhaus) and disposed in staggered rows, each pot corresponding to one of the 96 TOU-A accessions. On January 17, 2013 (day 0), a minimum of five *A. thaliana* seeds were sown in the central position of each pot. For all the treatments (with the exception of treatment A), the seeds for neighboring plants were evenly spaced, 2 cm away from *A. thaliana* in the central position (Figure 1). The germination rate of *A. thaliana* focal seedlings was daily monitored until 6 days after sowing. At this time, five accessions had a poor germination rate (between 0 and 6.25%) and were therefore discarded from further analyses. The plants that germinated after 6 days (i.e., 1.16%) were also discarded from further analyses.

The focal seedlings of *A. thaliana* and the neighboring seedlings were thinned to one per pot 18–20 days after seed sowing. The plants were grown at 20°C under natural light supplemented by artificial light to provide a 16 h photoperiod and were top watered without supplemental nutrients. The experiment lasted 87 days, from sowing to harvesting the last plants.

#### Measured Phenotypic Traits

Three raw phenotypic traits were measured on each focal plant of *A. thaliana* at the time of their flowering (FLO), which was measured as the number of days between the germination and flowering dates. The first trait corresponds to the height from the soil to the first flower on the main stem (H1F expressed in mm). H1F is related to seed dispersal (Wender et al., 2005) and shade avoidance (Dorn et al., 2000) in *A. thaliana*. The two other traits were used as proxies of resources accumulation. The maximum diameter of the rosette was measured at the nearest millimeter (DIAM; Weinig et al., 2006). This trait is a proxy of the growth of the rosette of the focal plant from germination to flowering. The above-ground dry biomass (BIOMASS, expressed in grams, with a precision down to one-tenth of a milligram) was estimated by drying the aboveground portion for 48 h at 60°C. We additionally quantified the strategy adopted by *A. thaliana* in response to the neighboring plants by calculating the ratio HD as the height of the first flower on the rosette diameter (i.e., H1F/DIAM). The values of HD below and above 1 would correspond to an aggressive and escape strategy, respectively (Baron et al., 2015).

Although all the plants bolted at the end of the experiment (i.e., 87 days after sowing), the plants that had not flowered at this time (i.e., 1.15%) were assigned a flowering date value of 87. The values of H1F and HD were therefore not available for these plants. All the raw phenotypic data are available in Supplementary Dataset 1.

### Statistical Analysis

#### Exploring Natural Variation of Plant-Plant Interactions at Different Levels of Complexities

Using the *lmer* function implemented in the *R* library *lmerTest* (Kuznetsova et al., 2017), the following mixed model was used to explore the genetic variation of response among the 91 TOU-A accessions (Methods in Supplementary Information):


$${\text{Y}}_{i\,j\,k\,l}=\mu_{t\,r\,a\,i\,t}+{\text{block}}_{i}+{\text{treatment}}_{j}+{\text{block}}_{i}\times {\text{treatment}}_{j}+{\text{accession}}_{k}+{\text{treatment}}_{j}\times {\text{accession}}_{k}+{\text{germ}}_{l}+\varepsilon_{i\,j\,k\,l}$$

where “Y” is one of the phenotypic traits scored on focal *A. thaliana* plants, “μ” is the overall phenotypic mean; “block” accounts for the differences in micro-environment among the four experimental blocks; “treatment” corresponds to the effect of the 12 treatments (A,AAA,PPP,PPS,PPV,PSS,PSV,PVV,SSS,SSV,SVV, and VVV); “accession” measures the effect of the 91 accessions; the interaction term “treatment × accession” accounts for genetic variation in reaction norms across the 12 treatments; the term “germ” is a covariate accounting for natural variation for the germination date among the 91 accessions; and “ε” is the residual term. All the factors were treated as fixed effects because the levels of no factor were the random samples from a population to which we intended to extrapolate. Given the split-plot design used in this study, the only exception was the “block × treatment” term that was treated as random, thereby allowing to use its variance as the error term for testing the “block” and “treatment” effects. The residual variance of the term “ε” was used for the calculation of the *F*-values for the terms “accession” and “treatment × accession.”

For each treatment, the estimated marginal means (EMM) were obtained for each accession using the function *emmeans* implemented in the *R* library *emmeans* (Lenth et al., 2018) (Methods in Supplementary Information). Because *A. thaliana* is a highly selfing species (Platt et al., 2010), EMM corresponds to the genotypic values of accessions.

To test whether the five phenotypic traits were not redundant, we estimated for each treatment the genetic correlation for each pair of traits, by calculating the correlation coefficient of Pearson based on the genotypic values.

Using the function *marker_h2_means* implemented in the *R* library *heritability* (Kruijer et al., 2016), the narrow-sense heritability of each phenotypic trait (*h*^2^_trait_) was estimated by fitting the genotypic values of accessions against the corresponding kinship matrix estimated on the whole set of 1,692,194 SNPs detected among the 91 accessions (Methods in Supplementary Information). This set of SNPs was obtained from the whole-genome sequencing of 195 accessions of the TOU-A population that included the 91 accessions used in this study (Frachon et al., 2017).

#### Detecting the Presence of Crossing Reaction Norms

Crossing reaction norms requires that (i) different accessions show different slopes across the environments (with the extreme case of significant differences on the sign of the slope across two environments), and (ii) the ranking of accessions largely differs among environments. This pattern corresponds to the cross-over effect (either symmetrical or asymmetrical) (Lacaze et al., 2009). To estimate the strength of crossing reaction norms for each phenotypic trait, we estimated the across-environment genetic correlations for each pairwise treatment combination by calculating the correlation coefficient of Pearson based on the genotypic values (*cor.test* function implemented in the *R* environment). Significant crossing reaction norms were detected by testing whether 95% confidence intervals of Pearson’s *r* was not overlapping with the value of 1.

### Genome-Wide Association Mapping Combined With a Local Score Approach

The effects of population structure on phenotype-genotype associations have been demonstrated to be limited in the TOU-A population (Brachi et al., 2013; Baron et al., 2015). Nevertheless, GWA mapping was run using a mixed-model approach implemented in the software genome-wide efficient mixed model association (GEMMA, Zhou and Stephens, 2012). This model includes a genetic kinship matrix as a covariate to control for the effect of the demographic history of the TOU-A population. In this study, we discarded SNPs with more than 17 missing values across the 91 accessions. In addition, because rare alleles may lead to an inflation of low *p*-values (Atwell et al., 2010; Brachi et al., 2010), we only considered the SNPs with a minor allele relative frequency (MARF) > 10%, leaving us with 630,234 SNPs. GEMMA was run independently on H1F, DIAM, HD, and BIOMASS for each of the 12 treatments. H1F, DIAM, HD, and BIOMASS were genetically correlated with FLO (mean value ± SEs across the 12 treatments, BIOMASS: Pearson’s *r* = 0.768 ± 0.043, DIAM: Pearson’s *r* = 0.360 ± 0.133, H1F: Pearson’s *r* = − 0.434 ± 0.085, HD: Pearson’s *r* = − 0.625 ± 0.015). The FLO genotypic values were thus included as a covariate in the GEMMA analyses, thereby allowing controlling for the putative effects of a confounding developmental stage (Uffelmann et al., 2021).

Thereafter, we implemented a local score approach on the set of *p*-values provided by GEMMA. The local score allows accumulating the statistical signals from contiguous genetic markers to detect significant genomic regions associated with phenotypic natural variation (Fariello et al., 2017). In each QTL region, the association signal, through the p-values, will cumulate locally due to linkage disequilibrium between the SNPs, which will then increase the local score (Bonhomme et al., 2019). The tuning parameter ξ was fixed at 2 (Bonhomme et al., 2019). Significant SNP-phenotype associations were identified by estimating a chromosome-wide significance threshold for each chromosome (Bonhomme et al., 2019).

### Identification of Candidate Genes Associated With Response to the Monospecific and Plurispecific Interactions

To identify the candidate genes associated with natural variation of plant-plant interactions, we retrieved genes around the significant zones identified by the local score approach. Using a custom script, we selected all genes inside the zones as well as the first gene upstream and the first gene downstream of these zones. The TAIR 10 database^2^ was used as our reference, leaving us with 4,524 unique candidate genes (*N*_BIOMASS_ = 1,538; *N*_DIAM_ = 1,008; *N*_H1F_ = 1,559; and *N*_HD_ = 1,572; Supplementary Figure 2).

In order to identify significantly over-represented biological processes (*P* < 0.05) in response to the different neighborhoods for the different traits, we submitted the candidate gene lists to the classification superviewer tool (^3^ Provart and Zhu, 2003) using the MAPMAN classification.

## Results

### The Extent of Natural Genetic Variation of Response to the Bispecific and Plurispecific Interactions

Highly significant genetic variation was found across the 12 treatments (Figure 1) for the five phenotypic traits scored on the focal *A. thaliana* plants (Table 1 and Supplementary Table 1). Similar results were observed without considering the treatments where *A. thaliana* was grown alone or with clones (Supplementary Tables 2, 3). The narrow-sense heritability SNP-based estimates were significant for 96.7% of the 60 “phenotypic trait × treatment” combinations (Supplementary Table 4) and ranged from 0.36 to 0.99 (mean = 0.72 and median = 0.76), suggesting that a large fraction of the phenotypic variation observed within each treatment was driven by genetic differences among the local *A. thaliana* accessions (Figure 1).

**TABLE 1:** Natural variation of five phenotypic traits scored on *Arabidopsis thaliana* plants in the 12 treatments.

	Traits									
	
	FLO		BIOMASS		DIAM		H1F		HD	
					
Model terms	*F*	*P*	*F*	*P*	*F*	*P*	*F*	*P*	*F*	*P*
Block	4.93	**6.11E-03**	1.81	1.42E-01	2.63	6.60E-02	1.81	1.64E-01	1.57	2.15E-01
Germ	2.46	1.17E-01	0.38	5.37E-01	0.45	5.01E-01	0.33	5.67E-01	0.41	5.22E-01
Treatment	12.56	**7.21E-09**	686.62	**< 1E-272**	139.93	**1.22E-24**	1.84	8.55E-02	31.32	**2.45E-14**
Accession	247.51	**< 1E-272**	112.52	**< 1E-272**	67.52	**< 1E-272**	28.92	**< 1E-272**	49.76	**< 1E-272**
Treatment x Accession	1.98	**2.12E-44**	9.45	**< 1E-272**	3.94	**3.69E-183**	1.67	**8.74E-25**	1.42	**1.21E-12**

*The bold P-values indicate significant effects after a false discovery rate (FDR) correction. FLO: flowering time, DIAM: maximum diameter of the rosette, H1F: height from the soil to the first flower on the main stem, HD = H1F/DIAM, and BIOMASS: aboveground dry biomass.*

**FIGURE 2:**
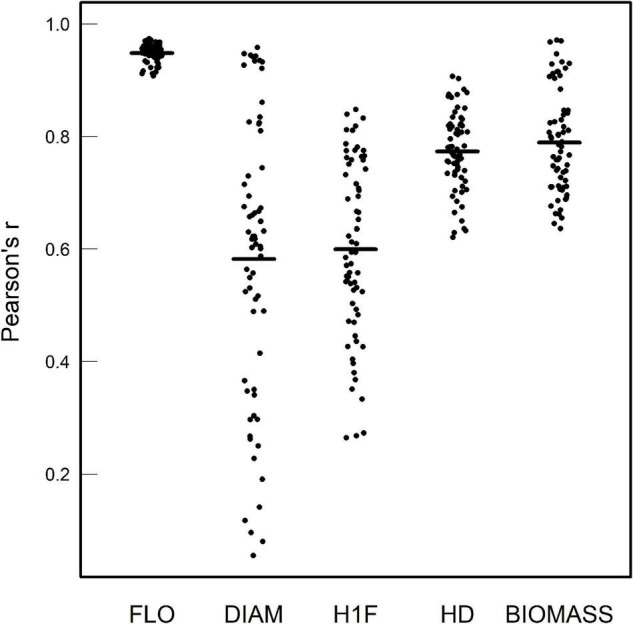
Pairwise genetic correlation coefficients of Pearson among the 12 treatments for each phenotypic trait. The dots correspond to the 66 pairwise treatment combinations. Black segments correspond to the mean value of Pearson’s *r*.

Importantly, as evidenced by highly significant “treatment × accession” interactions, a strong genetic variation of reaction norms was found for the five phenotypic traits, with or without considering the treatments where *A. thaliana* was grown alone or with clones (Table 1 and Supplementary Tables 2,3). For the phenotypic traits DIAM, H1F, HD, and BIOMASS, the across-environment genetic correlations ranged from 0.06 to 0.97 (mean = 0.69 and median = 0.73; Figure 2), indicating that the ranking of accessions for a given phenotypic trait largely differed among the 12 treatments. This pattern of crossing reaction norms is well illustrated for the height from the soil to the first flower for which the mean value was similar among the 12 treatments (Figure 1, Table 1, and Supplementary Table 1). In contrast, the across-environment genetic correlations for FLO were very close to unity (min = 0.91, max = 0.97, mean = 0.95, and median = 0.95; Figure 2), suggesting that the ranking of flowering time was similar among the 91 accessions across the 12 treatments. FLO was therefore discarded from further analyses.

For each treatment, the genetic correlations among the four remaining phenotypic traits were significantly different from the unity (absolute values of Pearson’s *r*: min = 0.02, max = 0.89, mean = 0.53, and median = 0.62), suggesting that the traits scored in this study partly behave independently (Supplementary Figure 3).

### Genetic Architecture Revealed by Genome-Wide Association Mapping Combined With a Local Score Approach

To compare the genetic architecture underlying the competitive response of *A. thaliana* between the bispecific and plurispecific neighborhoods, we combined GWA mapping based on 630,234 SNPs (i.e., 1 SNP every 189 bp) with a local score approach (Bonhomme et al., 2019). This combination of approaches was demonstrated to be efficient in the TOU-A population, allowing a fine description of the genetic architecture (in particular, the detection of QTLs with small effects) associated with quantitative disease resistance to the bacterial pathogen *Ralstonia solanacearum* and the cloning of four of the detected QTLs (Aoun et al., 2020; Demirjian et al., 2021).

The genetic architecture associated with natural variation of the traits DIAM, H1F, HD, and BIOMASS was polygenic with a number of detected QTLs ranging from 13 for BIOMASS in the SVV treatment to 72 for H1F in the A treatment (mean = 32 and median = 28) (Supplementary Table 5). Across the 12 treatments, the mean number of detected QTLs was not significantly different among DIAM, H1F, HD, and BIOMASS (Supplementary Table 5).

**FIGURE 3:**
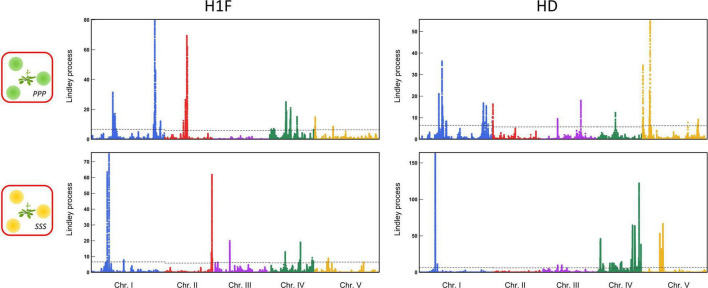
Identification of genomic regions associated with bispecific interactions in the TOU-A population. Manhattan plots of genome-wide association (GWA) mapping results for the PPP and SSS treatments for the “height from the soil to the first flower” (H1F, left-panel) and the ratio “height from the soil to the first flower/the rosette diameter” (HD, right-panel) traits. The *x*-axis indicates the physical position of the 630,234 single nucleotide polymorphisms (SNPs) along the five chromosomes. The *y*-axis indicates the Lindley process scores estimated from –log_10_ *p*-values from the mixed model implemented in the software genome-wide efficient mixed model association (GEMMA) using SNPs with a minor allele relative frequency (MARF) > 10%.

**FIGURE 4:**
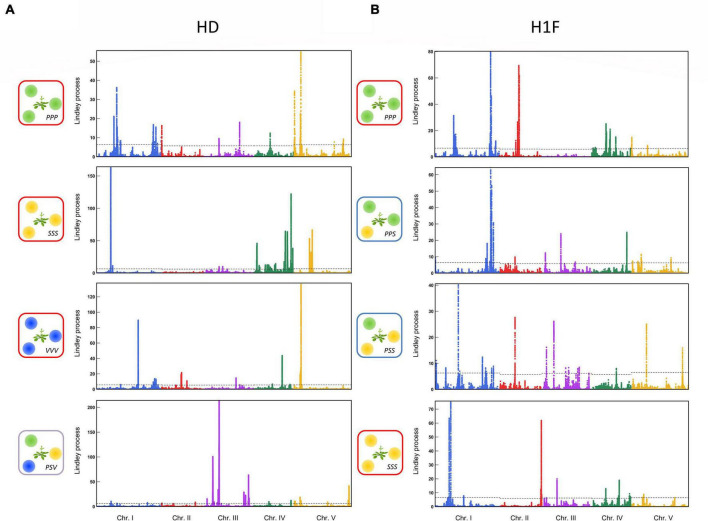
Comparison of the genetic architecture of response of *A. thaliana* among bispecific and plurispecific neighborhoods in the TOU-A population. **(A)** Manhattan plots comparing the GWA mapping results for the bispecific PPP, SSS, and VVV treatments and the plurispecific interaction treatment PSV for the HD ratio. **(B)** Manhattan plots comparing the GWA mapping results from the bispecific PPP to SSS treatments (PPP → PPS → PSS → SSS) for the H1F trait. The *x*-axis indicates the physical position of the 630,234 SNPs along the five chromosomes. The *y*-axis indicates the Lindley process scores estimated from -log_10_ *p*-values from the mixed model implemented in the software genome-wide efficient mixed model association (GEMMA) using SNPs with a minor allele relative frequency (MARF) > 10%.

For each phenotypic trait, the genetic architecture of the competitive response of *A. thaliana* was highly flexible among the 12 treatments. First, the genetic architecture of the competitive response of *A. thaliana* in the context of bispecific interactions was highly dependent on the identity of the neighboring species (Figure 3). For example, no significant QTL region was shared between the treatments PPP and SSS for H1F or between the treatments PPP and SSS for HD (Figure 3). Second, the genetic architecture of the competitive response of *A. thaliana* was highly dependent on the number of species co-occurring with *A. thaliana*. For instance, two very neat peaks of association were identified for HD in the middle of chromosome 3 in the treatment PSV (Figure 4A). While the first peak was not identified in the treatments PPP, SSS, and VVV, the second peak was identified in the treatment PPP but with a much lower association score (PSV: Lindley process value = 214 and PPP: Lindley process value = 9.5). Third, the genetic architecture of the competitive response of *A. thaliana* to the presence of a specific pair of neighboring species largely differed not only from the genetic architecture identified in the corresponding bispecific interaction treatments but also between the two related assemblages. As an illustration, we detected a neat association peak for H1F at the beginning of chromosome 1 in the treatment PSS (i.e., one *P. annua* individual + two *S. media* individuals), but neither in the treatment PPS (i.e., two *P. annua* individuals + one *S. media* individual) nor in the treatments PPP and SSS (Figure 4B).

**FIGURE 5:**
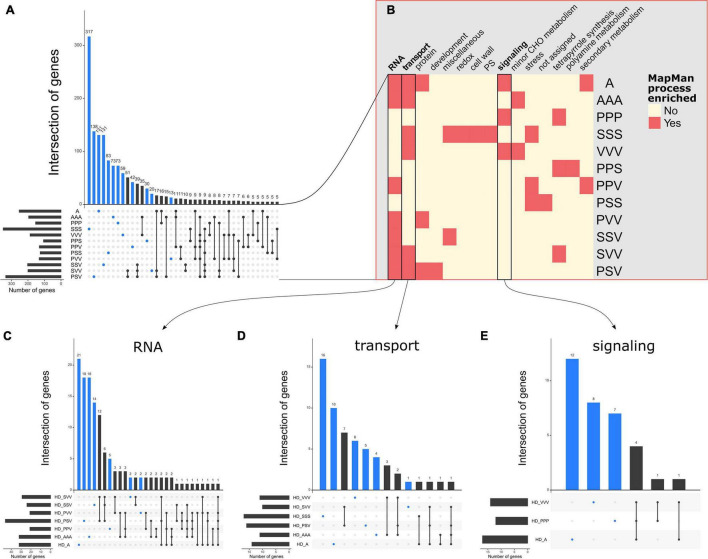
Illustrations of the flexibility of genetic architecture and the processes involved in HD among the 12 treatments. **(A)** An UpSet plot illustrating the variability of genes underlying the quantitative trait loci (QTLs) related to HD among the 12 treatments. **(B)** A heatmap illustrating the different MapMan enriched processes among the 12 treatments. **(C)** An UpSet plot illustrating the variability of genes involved in the RNA process underlying the seven treatments enriched in the RNA process. **(D)** An UpSet plot illustrating the variability of genes involved in the transport process underlying the six treatments enriched in the transport process. **(E)** An UpSet plot illustrating the variability of genes involved in the signaling process underlying the three treatments enriched in the signaling process. The blue dots and bar plots highlight the genes that are specific to only one treatment. UpSet plots were drawn with the *R* package *UpSetR* (Conway et al., 2017).

From these QTLs, we retrieved 4,524 unique genes located within and in the vicinity of 1 kb of the significant QTL regions detected for each “phenotypic trait × treatment” (Supplementary Dataset 2). In agreement with the values of genetic correlations between these four traits (Supplementary Figure 3), a large fraction of candidate genes was specific to a given treatment (BIOMASS: 77.4%, DIAM: 63.4%, H1F: 77.2%, and HD: 70.6%), suggesting that the genetic bases are largely distinct among the 12 treatments (Figure 5A and Supplementary Figure 4). Therefore, in this study, the genetic architecture underlying intraspecific variability of the competitive response largely depends on both the composition and assemblage of the neighborhood of *A. thaliana*.

### Identification of Enriched Biological Processes

Across the 48 “phenotypic trait × treatment” combinations, we identified 139 significantly over-represented gene ontology (GO, MapMan functional annotation) terms, which belong to 25 unique GO processes (Supplementary Dataset 3). Interestingly, the RNA related processes correspond to the most abundant GO term over-represented found in 28 “phenotypic trait × treatment” combinations, with eight treatments for BIOMASS, seven treatments for DIAM and HD, and six treatments for H1F (Figure 5, Supplementary Figure 5, and Supplementary Dataset 3). The second most represented GO terms are transport and minor CHO metabolism processes with 13 and 12 “phenotypic trait × treatment” combinations, respectively (Figure 5, Supplementary Figure 5, and Supplementary Dataset 3), but with different distribution across the traits. On one hand, the transport processes were represented in the four traits, while minor CHO metabolism processes were not found for BIOMASS in any of the 12 treatments. Conversely, the C1-metabolism processes were mostly enriched for BIOMASS in six treatments out of the seven “phenotypic trait × treatment” combinations identified for these processes (Supplementary Figure 5). The remaining enriched MapMan processes are related to tetrapyrrole synthesis, miscellaneous, protein, redox, secondary metabolism, stress, lipid metabolism, hormone metabolism, cell, photosystem, signaling, and not assigned genes (Figure 5, Supplementary Figure 5, and Supplementary Dataset 3).

## Discussion

What are the causes and consequences of variation in competitive ability is a long standing question in the studies on the dynamics of structure and diversity of plant communities (Aarseen, 1992). Some experimental and theoretical studies suggest that intraspecific genetic variation in competitive response and effect in a heterospecific context can promote maintenance of within-species genetic variation and species coexistence at a fine spatial scale (Aarseen, 1992; Fridley et al., 2007). This may originate from the rank order of competitive ability among the genotypes/species shifting from transitive to intransitive, depending on the identity of the genotypes of both the species (Bossdorf et al., 2009; Baron et al., 2015). On the other hand, other theoretical studies predict that variation among genotypes may negatively affect species coexistence. For instance, an extension of intraspecific niche variation due to genetic variation in the resources requirements would reduce niche differentiation among species (Hart et al., 2016). In addition, plant-plant interactions might constrain the life history evolution of a focal species, thereby limiting its evolutionary potential to respond to other environmental challenges than plant-plant interactions (Wilson, 2014). While informative, most of these studies consider the interactions between two plants species. However, a focal species rarely interacts with only one neighboring species either in crop fields or in more natural environments (up to 89 species, Wilson et al., 2012). Therefore, the genetics of plant-plant interactions and their consequences on the dynamics of plant coexistence need to be studied in a community context. In this study, we compared the genetic architecture of the competitive response of *A. thaliana* between bispecific and plurispecific neighborhoods in a highly genetically polymorphic local population of *A. thaliana* known to interact *in situ* with the three neighboring species considered in this study.

Several studies reported extensive genetic variation of the competitive ability of *A. thaliana* in the context of pairwise heterospecific interactions, both at the worldwide and local scales (Bossdorf et al., 2009; Brachi et al., 2012; Baron et al., 2015; Bartelheimer et al., 2015; Frachon et al., 2017, 2019; Palacio-Lopez et al., 2020). Based on several phenotypic traits related to resource accumulation and life-history traits, such as traits related to seed dispersal (Reboud et al., 2004; Wender et al., 2005), we also found an extensive local genetic variation of the competitive response of *A. thaliana* in all the plurispecific neighborhoods tested in this study. More importantly, we detected strong crossing reaction norms not only among the three bispecific interaction treatments but also among the different plurispecific neighborhoods surrounding the focal *A. thaliana* accessions. Altogether, these results suggest that the simultaneous interactions of *A. thaliana* with several plant partners can promote maintenance of the high genetic diversity observed in the TOU-A local population (i.e., only 5.6 times less than observed in a panel of 1,135 worldwide accessions) (Frachon et al., 2017). This diversity may in turn confer a high potential for *A. thaliana* to respond to the future modifications of the assemblages in the TOU-A plant community.

Testing whether the presence of genetic variation in competitive response in the plurispecific neighborhoods can promote species coexistence in the TOU-A plant community, would require several additional phenotyping and analyses. For instance, it would be interesting to test for the presence of genetic variation in the competitive effect of *A. thaliana* on the diverse competitor assemblages used in this study, thereby allowing testing whether a potential shift between transitivity and intransitivity depending on the identity of the accession of *A. thaliana* could vary between the bispecific and plurispecific neighborhoods. In addition, while we analyzed separately the phenotypic traits measured in this study, it may be worthwhile to analyze them simultaneously to test whether the direct and indirect gradients of selection acting on the phenotypic traits depend on the number of competitor species and/or plant assemblage (Baron et al., 2015; Palacio-Lopez et al., 2020). In the context of major ecological strategies (i.e., Grime’s Competitive-Stress-tolerant-Ruderal CSR theory; Grime, 1974, 1977), this may in turn help to test the importance of plasticity of trait syndromes in shifting the ecological strategies among the diverse plant assemblages (Takou et al., 2019). While *A. thaliana* is still considered a pioneer species with low competitive abilities and a typical ruderal strategy, several recent studies revealed that *A. thaliana* can inhabit highly competitive environments over several generations (Brachi et al., 2013; Bartoli et al., 2018; Frachon et al., 2019).

### A Flexible Genetic Architecture for Competitive Response Among the Bispecific and Plurispecific Neighborhoods

In agreement with the strong crossing reaction norms observed among the three bispecific interaction treatments, a GWA mapping approach revealed that the genetic architecture of *A. thaliana* competitive response in the bispecific neighborhoods was highly dependent on the identity of the neighboring species. Similar results were observed (i) in *A. thaliana* challenged with four neighboring species (including the three species used in this study) in field conditions (Baron et al., 2015) and (ii) in *Oryza sativa* challenged with the three weed species *Echinochloa oryzicola*, *Monochoria korsakowii*, and *Schoenoplectus juncoides* in greenhouse conditions (Onishi et al., 2018). Altogether, these results suggest that the effect of the identity of the neighboring species on the genetic architecture of competitive response is conserved among the focal plant species and across diverse phenotyping environments. The genetic architecture of *A. thaliana* competitive response was also highly flexible between the bispecific and plurispecific neighborhoods, suggesting that the genetic response of a particular accession of *A. thaliana* to a plurispecific neighborhood can be hardly predicted from the observation of its genetic responses in the corresponding bispecific neighborhoods. These results confirm our previous conclusions obtained from a GEA approach performed on 145 whole-genome sequenced natural populations of *A. thaliana* located in the southwest of France and described for various plant community descriptors (Frachon et al., 2019). Our results are also in line with previous studies reporting in *A. thaliana* (i) the limited overlap between QTLs underlying resistance to combined stress responses, such as drought plus the specialist herbivore *Pieris rapae*, and QTLs underlying resistance to the corresponding single stress response (Davila-Olivas et al., 2017; Thoen et al., 2017) and (ii) the distinct transcriptomic responses for single and combined stresses in diverse plant species, as illustrated for pathogen attack combined with heat stress (Suzuki et al., 2014; Desaint et al., 2021).

In this study, three non-exclusive and interconnected hypotheses can be proposed to explain the emergence of new QTLs in plurispecific neighborhoods. They are based on (i) the putative generation of new signals or elimination/modification of the pre-existing signals (e.g., light, aerial volatile organic compounds, root exudates, and nutrient availability) in the context of plurispecific interactions and (ii) their specific perception and subsequent signaling in *A. thaliana*. First, the amount of signals produced by the neighboring species A is strongly reduced in a plurispecific neighborhood, thereby leading to the strong reduction of the perception/signaling events activated in the context of bispecific interactions. Second, the production of new or modified signals in a given neighboring species is triggered by the presence of another neighboring species. Third, the simultaneous presence of different signals produced by the neighboring species A and B leads to the generation of new signaling processes. The two latter cases correspond to the production of new signals that emerged from higher-order interactions among the neighboring species (Levine et al., 2017).

### The Biological Pathways and Candidate Genes Associated With Competitive Response Depend on the Identity and Assemblage of Neighboring Species

While our experimental design identified a large set of genes (4,524 genes), we clearly observed an RNA process as a significant enriched biological process for most of the “phenotypic trait × treatment” combinations investigated, such as RNA processing, RNA binding, and a large variety of transcription factors, with a more pronounced proportion of BHLH and homeobox genes. These transcription factors are known to regulate multiple developmental processes, such as stress and light response (Bürglin and Affolter, 2016; Guo et al., 2021), and might exert a crucial role in promoting plant adaptation to diverse plant environments.

The second major over-represented biological process is related to the transport functions. Interestingly, 14 out of the 79 transport related proteins, identified in the seven treatments for ratio HD corresponding to the height of the first flower divided by rosette diameter, correspond to ABC (ATP-binding cassette) transporter proteins (Supplementary Dataset 2). Although none of them have been functionally characterized, these transporters have been recently proposed as key players of plant adaptation to their abiotic or biotic environment (Hwang et al., 2016). Interestingly, different ABC transporter genes were identified in the case of the bispecific interactions (ABC-B2 and G13) as compared with the plurispecific interactions (ABC-C3, C6, C7, and G26). Because of their diverse substrate specificities, they might constitute the essential components of perception/signaling pathways activated during plurispecific plant-plant interactions. Among the transport processes, nutrient foraging might also be a major response strategy in the context of plurispecific plant-plant interactions (Pierik et al., 2013).

Our observations, if considering all the enriched biological processes found in this study, are partially in agreement with the previously reported seven categories of functions identified in the artificial environments simulating plant-plant interactions: photosynthesis, hormones, cell wall modification and degradation, defense against pathogens, ABC transporters, histone modification, and meristem identity/life history traits (Subrahmaniam et al., 2018). However, transcriptional regulation appears here as a major previously unidentified process, in line with a highly flexible genetic reprogramming in response to the different plant assemblage environments.

## Conclusion

Our results suggest that plants can integrate and respond to different species assemblages depending on the identity and number of each neighboring species, through a large range of genes associated mainly with transcription adjustment (RNA process) leading to developmental and stress responses. Our experiment highlights that intraspecific variation in response to plant induced constraints is mainly due to processes not proposed to be involved in plant-plant communications (i.e., light, nutrients, volatile organic compounds, and root exudates). Complementarily to our findings in our GWA study, the transcriptomic and proteomic analyses of *A. thaliana* plants exposed to the bispecific and plurispecific neighborhoods would help to identify genes and proteins that are differentially regulated under these conditions. To our knowledge, no such studies comparing the global changes in protein and gene expression between the bispecific and plurispecific neighborhoods have been reported so far (Subrahmaniam et al., 2018). Another step to understanding the mechanisms underlying natural variation of plant-plant competitive responses would be (i) to functionally validate the identified candidate genes. This would open the way to functional analyses to investigate (ii) the nature of the signals perceived by the plant, and (iii) decipher the signaling pathways resulting from signal perception, leading to the plant response.

## Data Availability Statement

The original contributions presented in the study are included in the article/Supplementary Material, further inquiries can be directed to the corresponding authors.

## Author Contributions

FR, LA, and DR supervised the project. EB, LA, and FR designed the experiments. EB and JL conducted the greenhouse experiment and measured the phenotypic traits. CL analyzed the phenotypic traits, performed the GWA mapping, and performed and analyzed the enrichment tests. CL, DR, and FR wrote the manuscript. All the authors contributed to the revisions.

## Conflict of Interest

The authors declare that the research was conducted in the absence of any commercial or financial relationships that could be construed as a potential conflict of interest.

## Publisher’s Note

All claims expressed in this article are solely those of the authors and do not necessarily represent those of their affiliated organizations, or those of the publisher, the editors and the reviewers. Any product that may be evaluated in this article, or claim that may be made by its manufacturer, is not guaranteed or endorsed by the publisher.

## Footnotes

1
http://www.herbiseed.com/home.aspx

2
https://www.arabidopsis.org/

3
http://bar.utoronto.ca/ntools/cgi-bin/ntools_classification_superviewer.cgi
