# Supplementary material for: The Genomic Architecture of Competitive Response of Arabidopsis thaliana Is Highly Flexible Among Plurispecific Neighborhoods
Source: Front Plant Sci. 2021 Nov 25;12:741122. doi: 10.3389/fpls.2021.741122 (PMC8656689; doi:10.3389/fpls.2021.741122)
Supplement: Supplementary file 5 [file Data_Sheet_5.docx]

**Supplementary Information**

**The genomic architecture of competitive response of *Arabidopsis thaliana* is highly flexible among plurispecific neighborhoods**

**Cyril Libourel, Etienne Baron, Juliana Lenglet, Laurent Amsellem, Dominique Roby^*^ and Fabrice Roux^*^**

*** Correspondence:** Corresponding Authors: [fabrice.roux@inrae.fr](mailto:fabrice.roux@inrae.fr) & [dominique.roby@inrae.fr](mailto:dominique.roby@inrae.fr)

3 Supplementary Datasets

5 Supplementary Tables

6 Supplementary Figures

**Supplementary methods**

To identify the candidate genes underlying QTLs associated with 49 plant community descriptors (alpha-diversity, composition and abundance of the 44 most prevalent plant species) estimated for 145 natural plant communities inhabited by *A. thaliana* in south-west of France (Frachon et al., 2019), the corresponding GEA association results were first reanalyzed by applying a local score approach. This local score approach allows detecting significant genomic segments by accumulating the statistical signals from contiguous markers such as SNPs (Fariello et al., 2017). In a given QTL region, the association signal, through the *p*-values, will cumulate locally due to linkage disequilibrium between SNPs, which will then increase the local score (Bonhomme et al., 2019).

In order to apply this methodology on these GEA association results obtained from a Bayesian hierarchical model (Gautier 2015), we first ranked each SNP based on the Bayes Factor values obtained across the genome (from the highest to the lowest values) for each of the 49 plant community descriptors (Frachon et al., 2019). Then, each rank was divided by the total number of SNPs to obtain a *p* value associated with each SNP. The local score approach was then implemented on these *p* values to fine map genomic regions associated with plant community descriptors. The tuning parameter ξ was fixed at 2 (Bonhomme et al., 2019). Significant QTL regions were identified by estimating a chromosome-wide significance threshold for each chromosome (Bonhomme et al., 2019).

Candidate genes underlying significant QTL regions were retrieved using custom script developed under the *R* environment. An UpSet plot was set up in order to visualize in an efficient way the intersections of our 49 lists of candidate genes using the UpSetR package in R (Conway et al., 2017).

**R scripts to compute genotypic means and heritability tables**

library(stringr);library(dplyr);library(emmeans);library(lsmeans);library(phia);library(car);library(data.table);library(stargazer);library(lmerTest)

data = read.table("Dataset1.txt", h=T)

for(i in c(4:ncol(data))) {

data[,i] <- as.numeric(as.character(data[,i]))

}

*# for each trait (BIOMASS,DIAM, H1F, FLO and HD)*

mixed.lmer <- lmer(TRAIT ~ block + germ + treatment*accession + (1 | block:treatment), data = data, REML = TRUE)

emmeans = as.data.frame(emmeans(mixed.lmer, ~ accession | treatment))

Anova_model1 = as.data.frame(anova(mixed.lmer, type = "III", ddf = "Satterthwaite"))

emmeans =emmeans(mixed.lmer, ~ accession | treatment)

contrast(emmeans, interaction = "pairwise")

write.table(emmeans,"./TRAIT_EMMEANS_full_Model.txt",quote=F, col.names=T, row.names=F,sep="\t")

write.table(Anova_model1,"./output/TRAIT_Anova_full_Model.txt",quote=F, col.names=T, row.names=F,sep="\t")

*# without treatment A (alone)*

mixed.lmer <- lmer(TRAIT ~ block + germ + treatment*accession + (1 | block:treatment), data = data[data$treatment!="A",], REML = TRUE)

Anova_model1 = as.data.frame(anova(mixed.lmer, type = "III", ddf = "Satterthwaite"))

write.table(Anova_model1,"TRAIT_Anova_full_Model_without_A.txt",quote=F, col.names=T, row.names=F,sep="\t")

*# without treatments A and AAA*

mixed.lmer <- lmer(TRAIT ~ block + germ + treatment*accession + (1 | block:treatment), data = data[data$treatment!="A" & data$treatment!="AAA",], REML = TRUE)

Anova_model1 = as.data.frame(anova(mixed.lmer, type = "III", ddf = "Satterthwaite"))

write.table(Anova_model1,"TRAIT_Anova_full_Model_without_A_AAA.txt",quote=F, col.names=T, row.names=F,sep="\t")

*# estimating heritability values*

genotypic_means <- read.table("./TRAIT_EMMEANS_full_Model.txt",h=T)

list_id <- read.table("List_ecotype_id.txt",h=T)

kinship <- read.table("kin_matrix.kinf", h=T, check.names = F) # kinship was estimated from the .bed file from Frachon et al. 2017 using the following command: gemma -bfile genotypes.bed -gk 1 kin_matrix.kinf

rownames(kinship) <- kinship$ecotype_id

kinship <- as.matrix(kinship[,-1])

genotypic_means <- left_join(genotypic_means,list_id,by="accession")

Treatments = sort(unique(genotypic_means$treatment))

heritability_table = data.frame()

for (i in Treatments){

geno_means_treatment <- genotypic_means[genotypic_means$treatment==i,]

marker_h2_means = marker_h2_means(data.vector=geno_means_treatment$emmean, geno.vector=geno_means_treatment$ecotype_id, K = kinship, Dm=NULL, alpha = 0.05, eps = 1e-06,max.iter = 100, grid.size=99)

h2_estimators = data.frame(h2=round(marker_h2_means$h2,2), conf.int=paste0("(",round(marker_h2_means$conf.int1[1],2)," - ",round(marker_h2_means$conf.int1[2],2),")"), treatment=i)

heritability_table = rbind(heritability_table,h2_estimators)

}

write.table(heritability_table,"TRAIT_marker_h2_means.herit",quote=F, col.names=T, row.names=F,sep="\t")

**
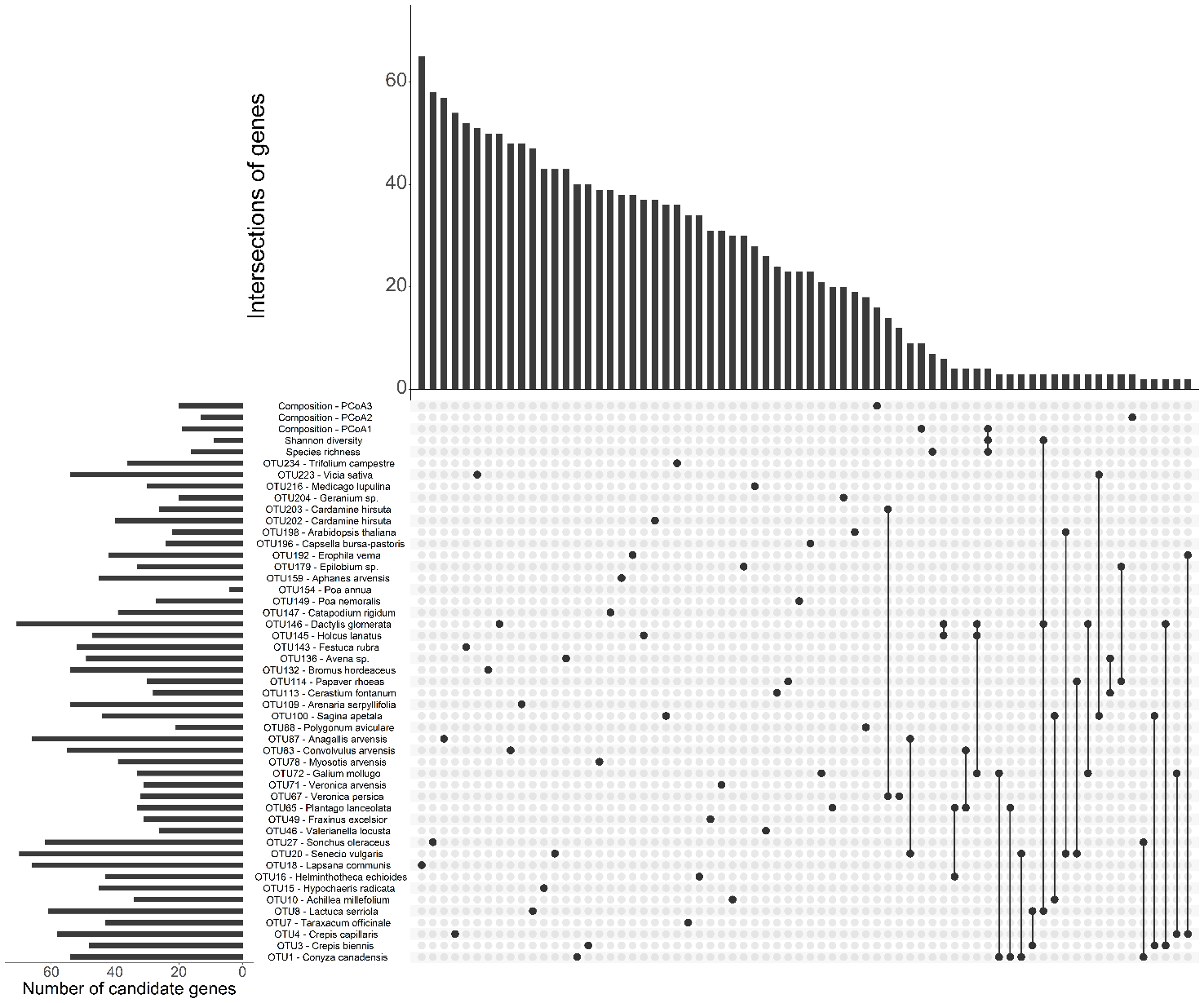
**

**Supplementary Figure 1. An UpSet plot showing the intersections of the 49 lists of candidate genes, each list corresponding to one of the 49 plant community descriptors characterized for 145 natural populations of *A. thaliana* located in south-west of France (Frachon et al., 2019).** Alpha-diversity of plant communities was approximated by estimating species richness and Shannon index. Plant community composition was approximated by the three first axes of a Principal Coordinate Analysis (PcoA) run on the abundance matrix of the 44 most prevalent plant species (i.e. species present in more than 10 populations).


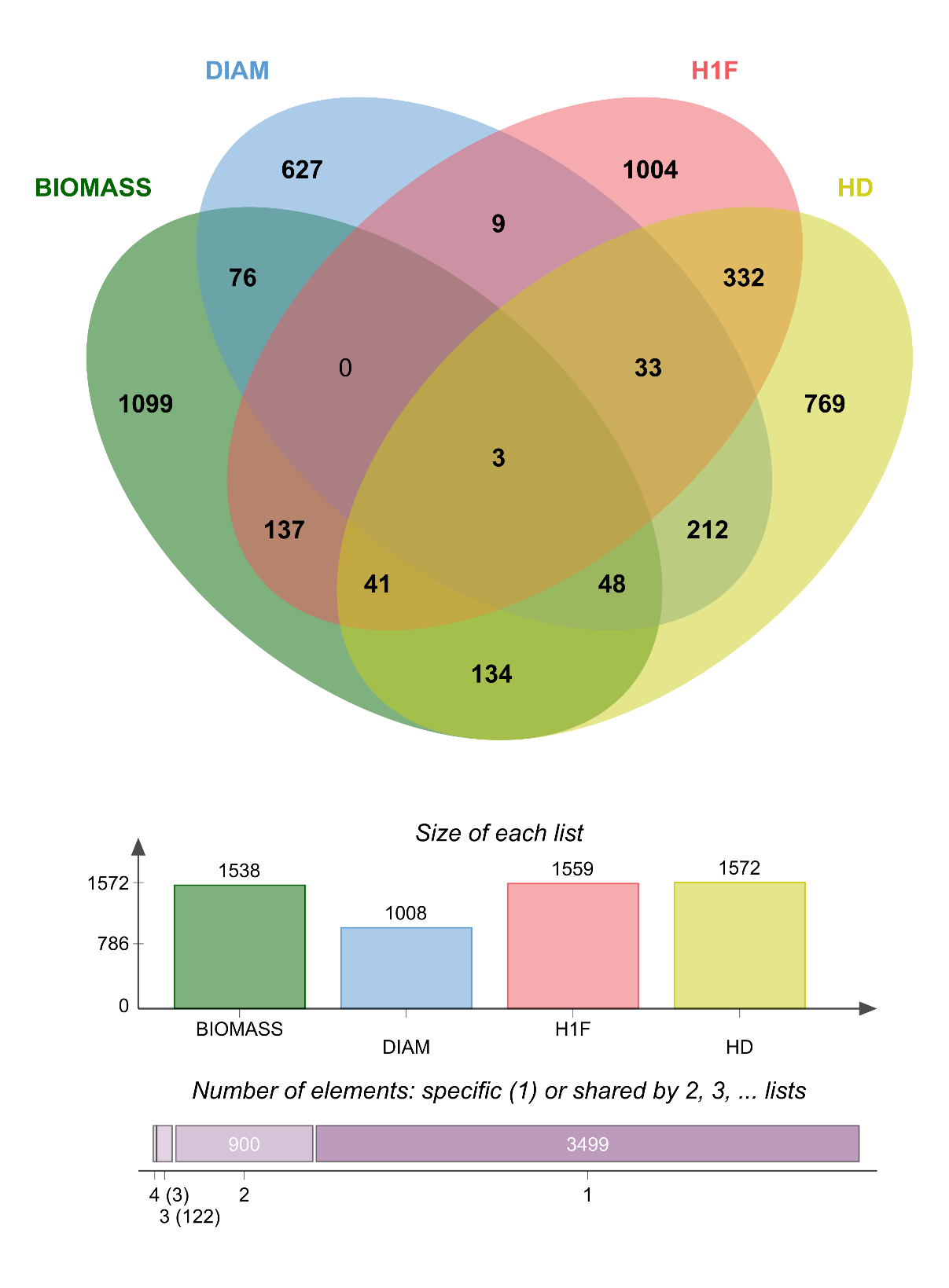


**Supplementary Figure 2. Non-proportional Venn diagram presenting the partitioning of candidate genes among the four phenotypic traits DIAM, H1F, HD and BIOMASS across the 12 treatments.** The Venn diagram was drawn using the *jvenn* online plug-in (Bardou et al., 2014).


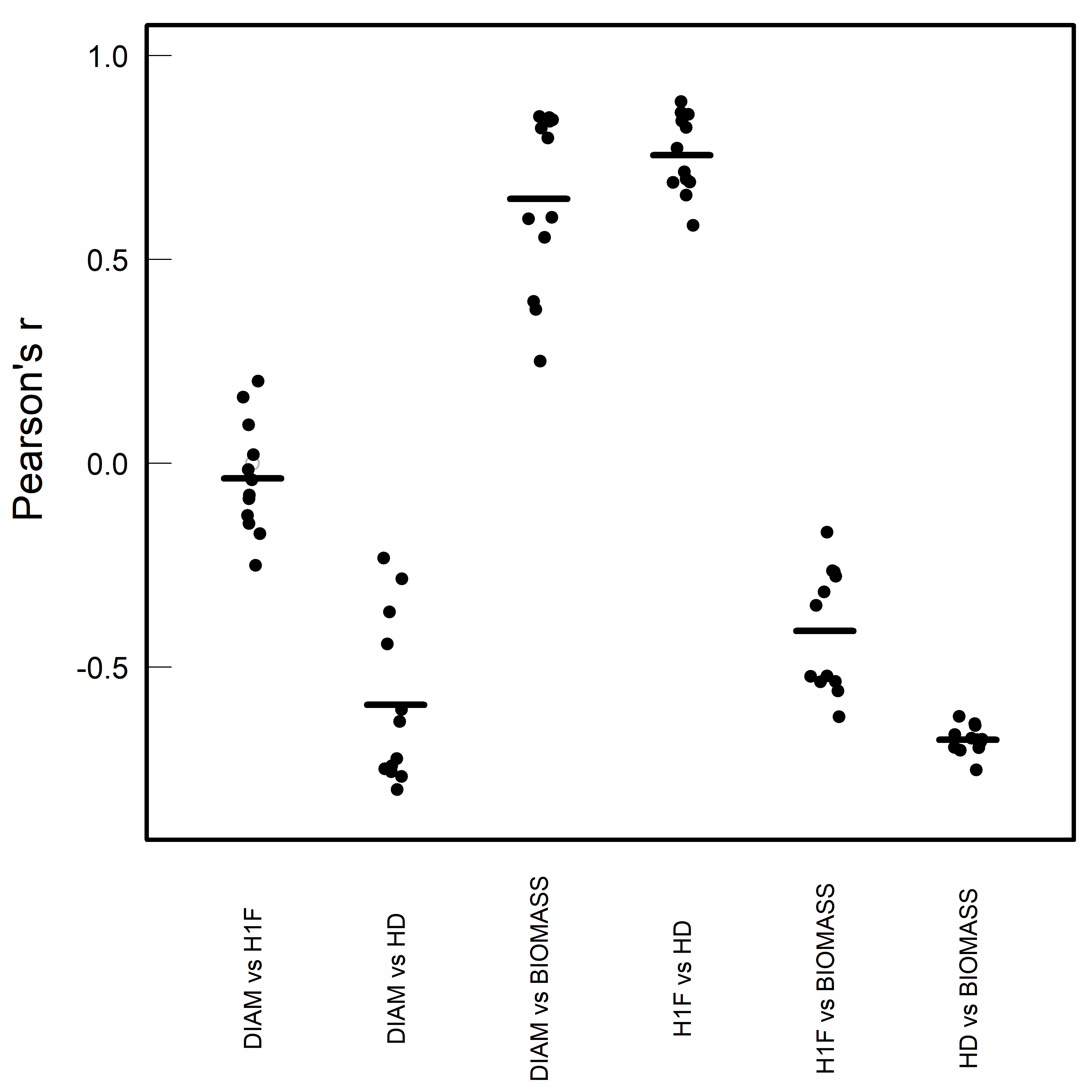


**Supplementary Figure 3. Pairwise genetic correlation coefficients of Pearson among the phenotypic traits DIAM, H1F, HD and BIOMASS for each treatment.** The dots for each pair of phenotypic traits correspond to the 12 treatments.


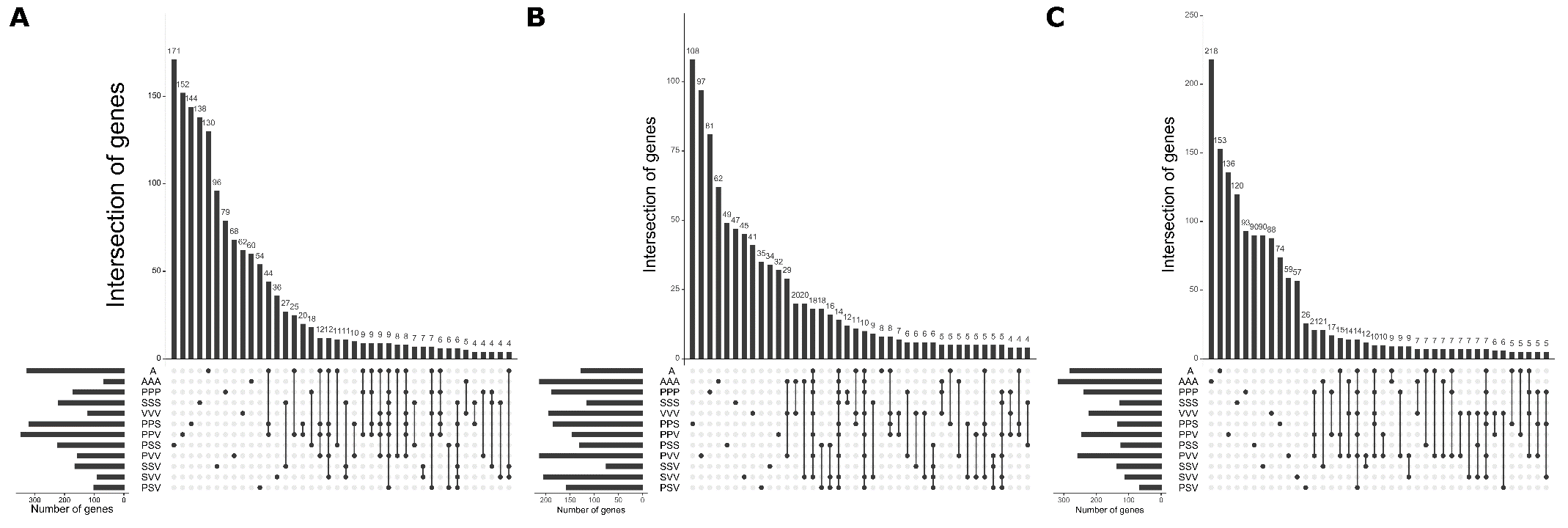


**Supplementary Figure 4. UpSet plots illustrating the flexibility of genetic architecture among the 12 treatments for BIOMASS (A), DIAM (B) and H1F (C).** Each list of candidate genes corresponds to one of the 12 treatments.

**
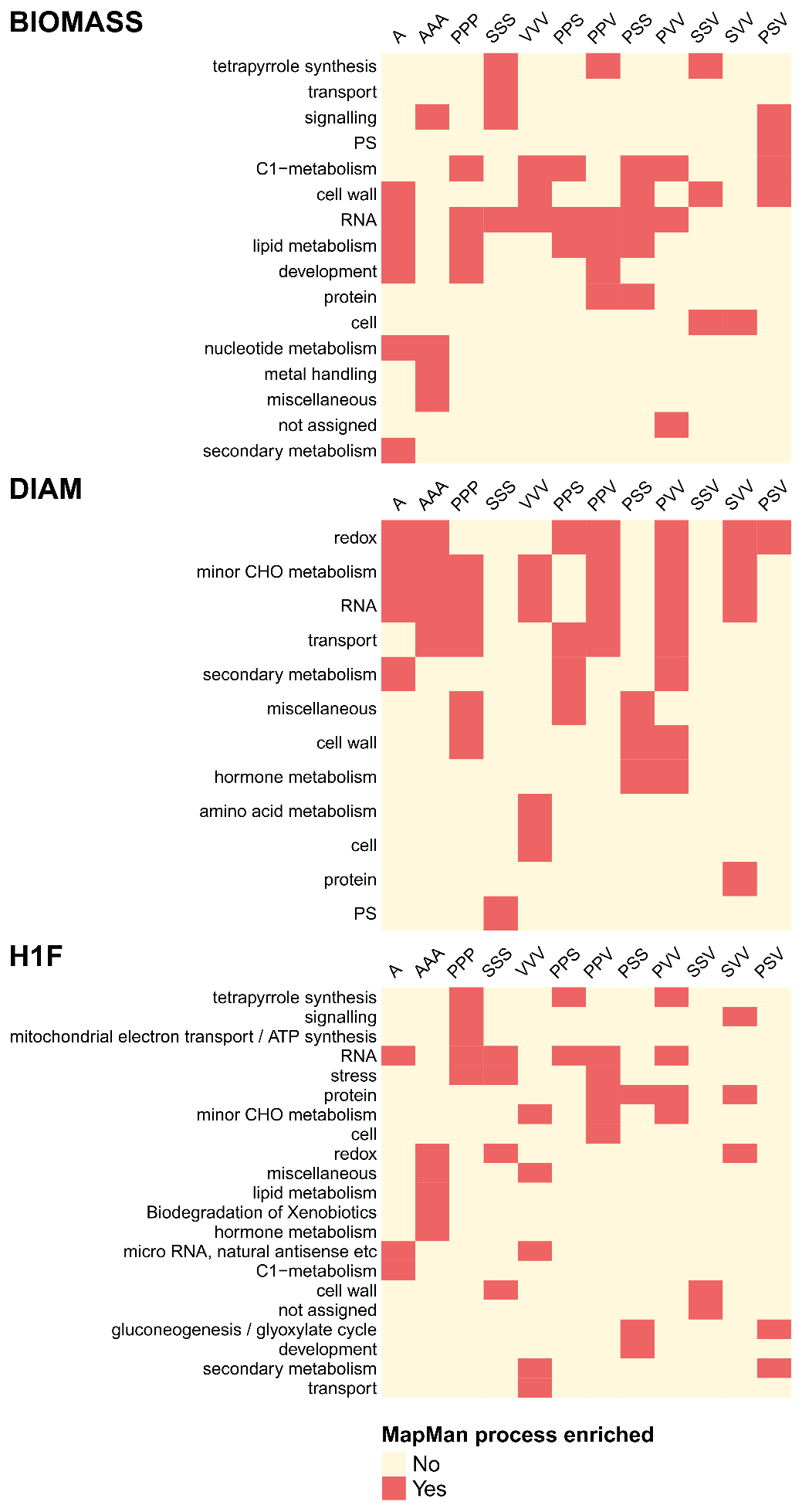
**

**Supplementary Figure 5. Heatmap illustrating the different MapMan enriched processes among treatments for BIOMASS, DIAM and H1F.**

**Supplementary Table 4. Narrow-sense heritability SNP-based estimates *h²* for the 60 ‘phenotypic trait * treatment’ combinations.** CI: 95% confidence intervals. Italic values indicate non-significant *h²* estimates. FLO: flowering time, DIAM: maximum diameter of the rosette, H1F: height from the soil to the first flower on the main stem, HD = H1F / DIAM, BIOMASS: aboveground dry biomass.

**Supplementary Table 5. Number of QTLs and number of candidate genes identified by GWA mapping combined with a local score approach for each ‘phenotypic trait * treatment’ combination.** DIAM: maximum diameter of the rosette, H1F: height from the soil to the first flower on the main stem, HD = H1F / DIAM, BIOMASS: aboveground dry biomass. No significant differences in the mean number of detected QTLs was observed among the four traits after applying of a Bonferroni correction (paired t-test; DIAM *vs* H1F: t = -2.75, *P* = 0.0188; DIAM *vs* HD: t = -2.45, *P* = 0.0326; DIAM *vs* BIOMASS: t = -0.93, *P* = 0.3713; H1F *vs* HD: t = 0.81, *P* = 0.4361; H1F *vs* BIOMASS: t = 1.70, *P* = 0.1178; HD *vs* BIOMASS: t = 1.44, *P* = 0.1765)
